# Use of a Community Engagement and Research Core model to enhance primary care provider recruitment: A case example

**DOI:** 10.1017/cts.2022.505

**Published:** 2022-11-25

**Authors:** Heidi Rishel Brakey, Julia M. Martinez, Andrew L. Sussman, Magdalena M. McWethy, Justin Martinez, Carla N. Cordova, Julie G. Salvador

**Affiliations:** 1 Clinical and Translational Science Center, University of New Mexico Health Sciences, NM, USA; 2 Family and Community Medicine, Comprehensive Cancer Center, University of New Mexico Health Sciences, NM, USA; 3 Department of Psychiatry and Behavioral Sciences, University of New Mexico Health Sciences, NM, USA

**Keywords:** CTSA, rural, primary health care, participant recruitment, community-engaged research

## Abstract

Persistent barriers exist to engage rural providers in research and training. Provider shortages exacerbate these challenges, leading to a scarcity of time and limiting motivation to participate in research. We present application of an innovative engagement model to increase rural primary care provider participation in research. Using our Community Engagement and Research Core, we demonstrate that fundamental principles of training and expertise, attention to efficiency and multitasking, and commitment to community are important for addressing provider recruitment barriers. We encourage other Clinical and Translational Science Centers to provide similar services to their local investigators to enhance provider engagement in research.

## Introduction

Persistent barriers have been documented regarding engagement of primary care providers (PCPs) in research, especially those in rural settings, with many citing lack of time or interest and disruption of clinic flow or responsibilities [1]. However, engagement in research is a critical process to enhance clinical practice and ultimately improve health outcomes [2]. In New Mexico, rural areas experience provider shortages [3], which can lead to a scarcity of time and therefore lack of motivation to participate in these activities [1]. The geography of the state brings further limitations to participation of in-person activities: approximately half of its dispersed population lives in rural areas, and as the fifth largest state, it can take 5 hours to travel one direction to city hubs [4].

The University of New Mexico’s Clinical and Translational Science Center [5] developed the Community Engagement and Research Core (CERC) in response to a growing number of investigators requesting support in community engagement efforts, particularly the ability to meaningfully engage diverse communities in research activities across remote parts of the state. The purpose of this paper is to present an example of the application of the unique CERC model to recruit and engage rural PCPs in research through partnership between CERC and a research project aiming to increase enrollment of rural New Mexico PCPs to treat patients with opioid use disorder.

## CERC Model for Recruitment

The University of New Mexico’s Clinical and Translational Science Center’s CERC was developed in 2010 and maintains trained staff with expertise in the full spectrum of community-engaged research to support expansion of research throughout New Mexico. CERC serves as a conduit for bidirectional communication between communities and researchers and provides critical support for researchers to engage with rural and underserved communities throughout the state [6]. This approach ensures substantive community-stakeholder participation throughout the research process, thus enhancing successful enrollment and retention of participants.

Specifically, this team provides free consultation for community-engaged studies, but what makes it unique from its counterparts across the country is its fee-for-service [7]: investigators hire CERC on an hourly basis for recruitment, research coordination, project management and implementation, information dissemination, and other related activities. Specially, the CERC model has proven successful in recruiting rural participants into research because of the team’s expansive training and expertise, efficiency by multitasking across multiple studies, and commitment to community.

### Training and Expertise

Since inception, CERC has worked on 180 research projects and is staffed by five skilled research professionals with credentials from complementary health-related fields with many years of experience in health research and related activities. CERC completes and maintains core research competencies (e.g., human subjects protections). Additionally, the team seeks numerous additional training opportunities contributing to their collective knowledge of community-engaged research (e.g., community-based participatory research, mental health first aid, implicit bias, health communication theory, and mixed methods research). When study teams collaborate with CERC, they have immediate access to highly trained and qualified staff.

### Attention to Efficiency and Multitasking

Contracts with CERC range from a few hours for one task or receive support from multiple team members for numerous hours over the course of the project. As staff leave, CERC works behind the scenes to train replacements while projects continue without losing time or money due to turnover. Further, working with CERC ensures a team with a wide-reaching network across the state. Individuals share recruitment ideas, often drawing from past connections or experience. Additionally, CERC works on multiple projects simultaneously and often travel to various rural areas of the state and the team can combine efforts as they travel and recruit for numerous projects in one trip.

### Commitment to Community

With the large number of community-engaged studies and related activities in which CERC is involved, the team has extensive collective statewide community-specific knowledge. For example, one team member conducted stakeholder interviews in rural areas across New Mexico [8] and transfers that community-specific knowledge to other projects (e.g., knowing and respecting that community’s culture and its preferred communication methods or venues). Additionally, another team member’s sole purpose is to build and enhance long-term, bidirectional communications between researchers and community stakeholders by developing partnerships with local primary care clinics, community health councils, community health workers, and other health-related stakeholders [6]. These relationships and established trust increase CERC acceptance by communities when recruiting for research projects.

## Case Example: Application of the CERC Model to Meet Recruitment Goals

### Study Overview and Recruitment

New Mexico was the setting of a study examining the impact of an online, interactive education and support model to help rural PCPs start and expand medication-assisted treatment for opioid use disorder (described elsewhere[9]). With less than 10% of the approximate 2,000 PCPs in the state prescribing to at least 10 patients [3,10], there is much opportunity to expand this treatment. Therefore, a critical step for this project was recruiting PCPs to participate in the curriculum intervention and accompanying study – a task that is simple in concept but difficult in practice.

Despite high rates of opioid addiction [11], scarce local treatment options [12], and strong evidence in favor of PCP-delivered medication-assisted treatment [13], the study team struggled to recruit providers. Aside from general challenges of recruiting PCPs into research projects, this study posed additional barriers because of its focus on treating opioid use disorder using buprenorphine. There is ample research describing barriers to this treatment, including stigma toward persons with OUD, lack of confidence and knowledge providing this treatment, concerns with Drug Enforcement Agency audits, among others [14].

This study aimed to enroll 80 rural PCPs over 4 years. The team began recruitment November 2017, coordinated by a single full-time staff person within the study lead’s department. Efforts included faxing rural clinics, in-person and phone meetings, using various clinician listservs, attending conferences, partnering with a practice-based research network, and word of mouth. Providers who joined the study were eligible to receive monetary compensation for data collection activities and continuing education units for participation in the curriculum training intervention. These efforts, unfortunately, were not sufficient to meet recruitment goals as it moved into its second year. Recruitment difficulties the study team experienced are not uncommon. It is hard for investigators to establish and maintain connections with communities and find staff who have familiarity with best practices in community-engaged research [15]. In September 2018, the study team sought support for recruitment and turned to CERC to employ other engagement strategies from staff with established community engagement and recruitment experience.

### CERC Involvement

After a short initiation period, the CERC and study teams divided their efforts by role and function. CERC was primarily responsible for recruitment, enrollment, and data collection while the original study team concentrated on intervention implementation, funder reporting, and dissemination (Table 1). Throughout this process, the CERC and study teams worked closely together, meeting weekly to discuss recruitment efforts, challenges, and strategies.

**Table 1.** Primary responsibilities of the study team and CERC^*^

	Study team	CERC
**Primary responsibility**	• Administer intervention • Funder reporting • Dissemination	• Recruitment • Enrollment • Data collection

* Community Engagement and Research Core.

**Fig. 1.**
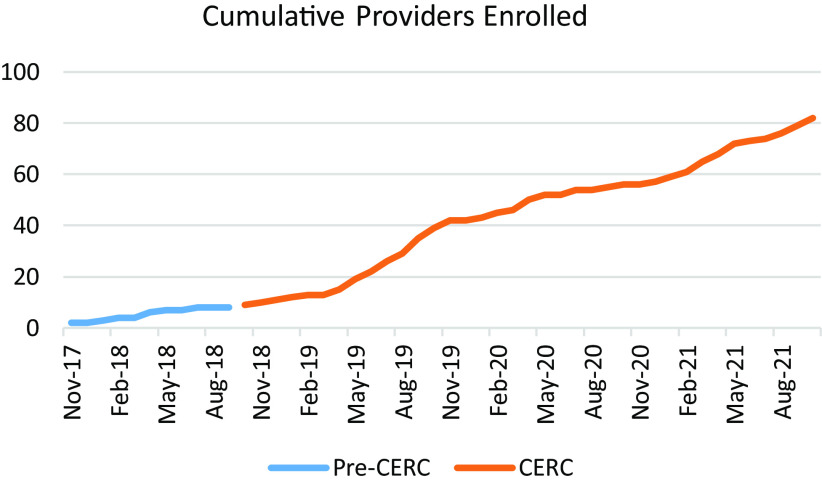
Number of cumulative providers enrolled into study before and after involving CERC*. *Community Engagement and Research Core.

*Impact on Recruitment.* Once the study team collaborated with CERC, recruitment went up threefold over 1 year (Fig. 1). Prior to CERC’s involvement, from November 2017 to September 2018, eight PCPs enrolled. Within 1 year after CERC joined the team (October 2018), 27 new PCPs joined the study and steadily increased until reaching the recruitment goal (*n* = 80) in October 2021.

### CERC’s Strategies for Success

Following a brief period of study orientation and review of previous recruitment methods, the CERC team revised strategies and applied their established model for recruitment of training and expertise, efficiency and multitasking, and commitment to community (Table 2).

**Table 2.** CERC^*^ model to promote recruitment as it applies to one study

Domains	Benefits to study
Training and expertise	• CERC staff with health and community-engaged research experience and training
Efficiency and multitasking	• CERC maintains sufficient staff (N = 5) with high retention • Team members able to cross-present projects • Use of existing networks • Present multiple projects to maximize resources
Commitment to community	• Adapt approaches to community context • Establish relationships and trust with partners

* Community Engagement and Research Core.

For an equivalent cost of one full-time study coordinator, CERC provided five team members to the study and immediately helped by sharing established connections and past recruitment successes. Throughout this partnership, as study team staff turnover occurred, they did not hire replacements and relied more on CERC’s services. As CERC staff turnover happened, the study continued seamlessly, as CERC trained their new staff behind the scenes, with no time lost or additional cost to the research project.

Prior to the COVID-19 pandemic, CERC relied heavily on in-person recruitment visits, including cross-promotion of multiple studies to enhance reach. This strategy expanded recruitment and maximized limited resources needed to connect with prospective providers and clinic sites throughout the state (e.g., associated travel expenses and staff time). Recruitment slowed during the first year of the pandemic (March 2020 to February 2021), as demonstrated in Fig. 1. Providers were facing numerous pandemic-related challenges [16,17], and CERC and the study team re-strategized recruitment techniques, relying more on virtual methods (e.g., phone calls, faxes, virtual presentations) and provider-to-provider recruitment. While CERC never traveled again for this study, the team was still able to enroll providers steadily.

With their knowledge and experience in community-engaged research, CERC did everything they could to make their efforts community centric. For example, the team knew of and used specific approaches to recruitment in specific communities (e.g., work with community gatekeepers prior to approaching clinics and not to dress “too city” in certain areas [8]). Additionally, CERC adapted to each clinic’s capacity and communication styles. Often, clinics would express they were unable to participate in the study due to time constraints, limited staff and resources, or perceived leadership priorities; CERC acknowledged, documented, and responded effectively. For example, at initial contact to one clinic, a representative asked for the team to reach out months later. CERC worked collaboratively and internally documented each encounter so everyone was aware of the clinic’s level of engagement and when to contact them again. With this, CERC was able to acknowledge and respect the clinic’s needs and interest.

Finally, to establish more bidirectional relationships, CERC disseminated study results back to participants and communities through health council presentations, demonstrating how participation in this study was impacting their community by increasing the number of prescribers providing medication-assisted treatment. The goal was to promote interest in the project and have PCPs join or share with others who may be interested in the study.

## Discussion

This article presents application of the CERC model to expand engagement of PCPs in research. Such efforts, especially among healthcare providers in rural settings, recognize their essential translational role to better understand how to adapt and implement clinical interventions to benefit communities [1]. The study team and CERC, however, experienced common challenges gaining provider interest to participate in research and/or to treat patients with opioid use disorder [1,14] including lack of time, interest, leadership support, and/or perceived patient need. These were exacerbated with the pandemic [16,17] and CERC experienced greater difficulty recruiting providers who had even less time, were more stressed and overworked, and did not want to integrate new clinical responsibilities. Despite these challenges, with CERC’s assistance, the study team has seen success in expanding its program to train numerous PCPs and expanding medication-assisted treatment across our rural state.

Based on our experience in this case study, we documented increased enrollment after implementing the CERC model and propose the model may be useful to enhance enrollment in other research projects. While individual recruitment methods described here are relatively common [18,19], the combination of these elements and the team’s dynamic interplay are innovative. This model demonstrates that core principles of training and expertise, attention to efficiency and multitasking, and commitment to community can address recruitment barriers. Key characteristics of CERC include multiple trained team members who can share duties and move seamlessly through staff turnover; a team working on multiple statewide studies can combine efforts; years of shared experience bringing established and longstanding connections and relationships statewide; and a commitment to and expertise in community-engaged methods making recruitment efforts more community- and person-centered.

Our Clinical and Translational Science Center counterparts across the country have developed several recruitment support mechanisms and resources to support investigators including recruitment feasibility assessments, consultation, planning, and budgeting; help with study advertisements and recruitment materials; and direct recruitment, use of electronic medical records, social media services, screening/scheduling, and direct connection to commercial recruitment support [18]. Only 28% of CTSC survey respondents in Niyibizi *et al*. [7] said they performed direct recruitment of participants, however. After an internet search, we were unable to determine whether other CTSCs employ a similar model. Some institutions cite availability of consultation, recruitment, and coordination services, but none were configured in the manner described here with this unique combination of bundled components including its fee-for-service model; expansive training, expertise, and experience; numerous statewide community relationships and already established trust; and ability to provide a high level of efficiency and cost saving to the study team.

### Limitations

We recognize this is a single case example not designed to attribute causation. Further, changes in application of the CERC model due to pandemic-related changes introduced variation and challenges that disrupted standard recruitment and engagement practices. However, given the consistent increases in enrollment upon implementation of this model coupled with the urgent need to identify promising practices to enhance recruitment of PCPs in research, we present these findings for consideration and recommend further examination using experimental methods to assess model components.

## Conclusion

The CERC model can be a useful approach to increase recruitment of PCPs in research through its highly trained research staff and team-based approach. Other CTSCs can provide similar services to their local investigators to help enhance primary care research engagement that can ultimately inform practice improvements.
